# Size dependent microbial oxidation and reduction of magnetite nano- and micro-particles

**DOI:** 10.1038/srep30969

**Published:** 2016-08-05

**Authors:** James M. Byrne, Gerrit van der Laan, Adriana I. Figueroa, Odeta Qafoku, Chongmin Wang, Carolyn I. Pearce, Michael Jackson, Joshua Feinberg, Kevin M. Rosso, Andreas Kappler

**Affiliations:** 1 Geomicrobiology, Center for Applied Geosciences, University of Tuebingen, Sigwartstrasse 10, 72076, Tuebingen, Germany; 2 Magnetic Spectroscopy Group, Diamond Light Source, Didcot, OX11 0DE, UK; 3 Williamson Research Centre for Molecular Environmental Science, School of Earth, Atmospheric and Environmental Sciences, University of Manchester, Manchester M13 9PL, UK; 4 Pacific Northwest National Laboratory, Richland, WA 99352, USA; 5 School of Chemistry, The University of Manchester, M13 9PL, Manchester, UK; 6 Institute for Rock Magnetism, University of Minnesota, 291 Shepherd Labs, 100 Union Street SE, MN 55455, Minneapolis, USA

## Abstract

The ability for magnetite to act as a recyclable electron donor and acceptor for Fe-metabolizing bacteria has recently been shown. However, it remains poorly understood whether microbe-mineral interfacial electron transfer processes are limited by the redox capacity of the magnetite surface or that of whole particles. Here we examine this issue for the phototrophic Fe(II)-oxidizing bacteria *Rhodopseudomonas palustris* TIE-1 and the Fe(III)-reducing bacteria *Geobacter sulfurreducens*, comparing magnetite nanoparticles (*d* ≈ 12 nm) against microparticles (*d* ≈ 100–200 nm). By integrating surface-sensitive and bulk-sensitive measurement techniques we observed a particle surface that was enriched in Fe(II) with respect to a more oxidized core. This enables microbial Fe(II) oxidation to occur relatively easily at the surface of the mineral suggesting that the electron transfer is dependent upon particle size. However, microbial Fe(III) reduction proceeds via conduction of electrons into the particle interior, i.e. it can be considered as more of a bulk electron transfer process that is independent of particle size. The finding has potential implications on the ability of magnetite to be used for long range electron transport in soils and sediments.

The ubiquity of Fe-bearing minerals in the environment has led to a number of bacteria developing pathways through which they can use Fe in different oxidation states as either electron donors or terminal electron acceptors^1^. On the one hand, this includes Fe(III)-reducing bacteria (e.g. *Geobacter sulfurreducens* or *Shewanella oneidensis*), which combine the oxidation of organic compounds or hydrogen with the reduction of minerals with short range order, such as ferrihydrite, often leading to the release of Fe^2+^_aq_ and the precipitation of siderite (FeCO_3_) and magnetite (Fe_3_O_4_), depending on conditions present during formation (e.g. pH, temperature, biomass concentrations and the presence of electron shuttles)^2^. On the other hand, there are three types of Fe(II)-oxidizing bacteria including nitrate-reducing bacteria (e.g. *Acidovorax sp* BoFeN1^3^), neutrophilic microaerophilic bacteria (e.g. *Gallionella ferruginea*^4^) and phototrophic Fe(II)-oxidizers (e.g. *Rhodopseudomonas palustris* TIE-1), which fix CO_2_ and oxidize Fe(II) in the presence of light^5^. Through these oxidative processes, minerals such as ferrihydrite, goethite and green rust are precipitated either externally or within their cell walls, with some evidence suggesting that magnetite can also be formed^6^.

To date, much of the Fe which is bioavailable to Fe(II)-oxidizing bacteria has commonly been considered to originate from aqueous or complexed Fe^2+^ 7. However, it has recently been shown that *R. palustris* can use a solid electrode as an electron donor^8^ and that the *c*-type cytochrome MtoA purified from a microaerophilic Fe(II)-oxidizing bacteria (*Sideroxydans lithotrophicus* ES-1) can oxidize the surface of magnetite and significantly change its stoichiometry^9^. Furthermore, it has also been shown that Fe(III)-reducers are able to use magnetite as terminal electron acceptors^10^,^11^,^12^. These observations were expanded in a recent study showing that magnetite in co-cultures is able to act as a recyclable electron donor or an electron acceptor, effectively a “biogeochemical battery”, to either Fe(II)-oxidizing and Fe(III)-reducing bacteria, respectively, depending upon ambient environmental conditions that affect the balance of microbial activity (i.e., visible light and acetate concentrations)^13^. Such processes are thought to enable the bacteria to use the magnetite to survive redox changes related to day and night cycles or fluctuations in water levels, such as those experienced by littoral sediments. Consequently, magnetite potentially plays an important role in environmental processes and more information is needed to clearly understand how characteristics of magnetite such as stoichiometry (i.e. Fe(II)/Fe(III) ratio) and particle size might influence this role. Furthermore, these processes may also have implications for the use of magnetite as a highly reactive remediation agent for treating polluted wastewater and soil^14^,^15^, as the reactivity of magnetite is highly dependent upon its surface stoichiometry, which in turn, could be inhibited by microbial processes^16^,^17^.

Magnetite is a mixed-valence material, based upon high-spin Fe^2+^ and Fe^3+^ ions in octahedral (Oh) and tetrahedral (Td) coordination according to the formula Fe^2+^_Oh_Fe^3+^_Td_Fe^3+^_Oh_O_4_^2−^. Fe^2+^-Fe^3+^ valence alternation electron exchange between adjacent Oh sites is fast at room temperature (~10^12^ s^−1^) enabling low bulk resistivity. The spin imbalance between the antiparallel Oh and Td iron sublattices leads to the ferrimagnetic properties of the mineral, as the magnetic moments of the octahedral site ions (Fe^2+^ and Fe^3+^) are aligned anti-ferromagnetically with the tetrahedral site ions (Fe^3+^), resulting in the moments of the ferric ions effectively cancelling each other out and leaving the net magnetic moment to be due to the Fe^2+^_Oh_ ions. Consequently, the magnetic properties of magnetite are highly sensitive to changes in stoichiometry. This is exemplified in the topotactically isomorphic mineral maghemite ((Fe^3+^_Td_[Fe^3+^_5/3_□_1/3_]_Oh_O_4_^2−^), which contains no Fe(II) and where □ represents a vacancy. Maghemite is known to have a lower bulk saturation magnetization (*M*_s_) of ~75 A m^2^ kg^−1^ at room temperature in comparison to the *M*_s_ = 92 A m^2^ kg^−1^ for stoichiometric bulk magnetite^18^. Several signature transition temperatures are evident for magnetite, including the isotropic point of pure magnetite at ~130 K^19^ and the Verwey transition (*T*_V_) which occurs at ~121 K and is a result of magnetite undergoing a first order phase transition from cubic crystal symmetry to a lower symmetry (likely monoclinic) structure^20^. *T*_V_ is known to be exceptionally sensitive to the presence of additional elements, e.g. Ti^4+^ 21 and Al^3+^ 22, as well as stoichiometry^23^, whilst its dependence upon size is less clear, especially in the superparamagnetic regime^24^.

Many questions remain about how Fe-metabolizing bacteria interact with magnetite, especially in their ability to use it as an electron donor or electron acceptor (i.e. biogeochemical battery). In particular, it is unclear to what extent the bacteria are able to access Fe in the whole magnetite particle, or whether such biogenic redox processes are only confined to the surface of the magnetite particle. To answer this, we have investigated the ability for separate cultures of Fe(II)-oxidizing and Fe(III)-reducing bacteria to use magnetite with significantly different particle sizes. Here we have incubated Fe(II)-oxidizing bacteria (*R. palustris*) and Fe(III)-reducing bacteria (*G. sulfurreducens*) with two distinct types of magnetite, nanoparticles (*d* ≈ 12 nm) and microparticles (*d* ≈ 100–200 nm) in order to test the effect that surface area to volume ratio has on the microbe-mineral electron transfer processes. We combine synchrotron-based, surface-sensitive x-ray magnetic circular dichroism (XMCD) with bulk sensitive ^57^Fe Mössbauer spectroscopy to probe the impact of both Fe(II) oxidation and Fe(III) reduction on the stoichiometry of the magnetite, with ancillary applied magnetic measurements and transmission electron microscopy (TEM) based imaging and spectroscopic information to further inform our findings.

## Results

### Magnetic susceptibility

**Figure 1:**
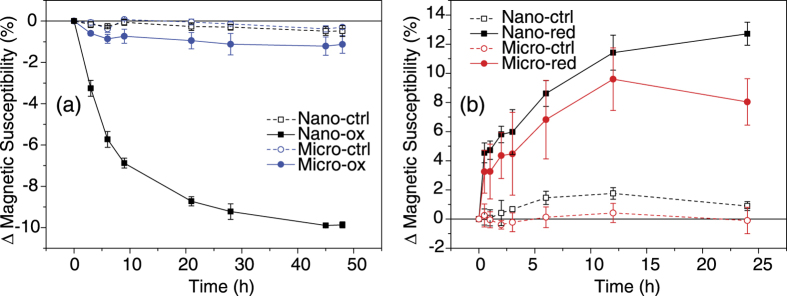
Relative change (%) in magnetic susceptibility from starting value (*t* = 0) during (**a**) oxidation (-ox) and (**b**) during reduction (-red) for both Nanomag and Micromag. Cultures were compared against control groups containing no bacteria (-ctrl).

Two types of magnetite were synthesized for these experiments, denoted Nanomag and Micromag in reference to their respective sizes, i.e. Nanomag corresponds to magnetite nanoparticles and Micromag corresponds to magnetite microparticles. Magnetic susceptibility (MS) measurements enable direct measurements of the magnetic changes to the microbe-magnetite cultures *in situ* without removing any sample (Fig. 1). From the MS measurements it was readily seen that the most substantial changes under Fe(II)-oxidizing conditions (Fig. 1a) occurred for the Nanomag, which showed a decrease in MS of 10% (from 703.5 ± 0.9 × 10^−6^ Système International d’Unités (SI) to 634.1 ± 1.7 × 10^−6^ SI), consistent with previous observations^13^, whereas Micromag showed a much smaller decrease of only 1% (from 726.5 ± 13.9 × 10^−6^ SI to 718.3 ± 10.7 × 10^−6^ SI). Application of the T-test indicated that differences between the relative changes of the MS of Nano-ox and Micro-ox were statistically different (i.e. *P* < *0.05*) at all time points. This result was expected due to the much higher surface area to volume ratio of the Nanomag compared to Micromag magnetite. However, changes under Fe(III)-reducing conditions are less clear (Fig. 1b). The MS increases by 12.7% for Nanomag relative to the starting value (from 724.6 ± 3.9 × 10^−6^ SI to 816.7 ± 2.6 × 10^−6^ SI) compared to 8.0% for Micromag relative to the starting value (from 757.9 ± 21.3 × 10^−6^ SI to 818.6 ± 11.8 × 10^−6^ SI). Furthermore, the differences between the two are not statistically significant with overlapping error bars until the final time-point measurement (24 h). The T-test showed that the relative changes of the MS of Nano-red and Micro-red were not statistically different from each other (i.e. *P* > *0.05*) at all time points until the final measurement. These data suggest that the surface area to volume ratio did not play a major role in the differences between Nanomag and Micromag reduction by *Geobacter sulfurreducens*. In all experiments, control groups showed some minor changes in MS, however these were determined to be statistically insignificant from setups containing bacteria.

### *Ex situ* mineralogical analysis

**Figure 2:**
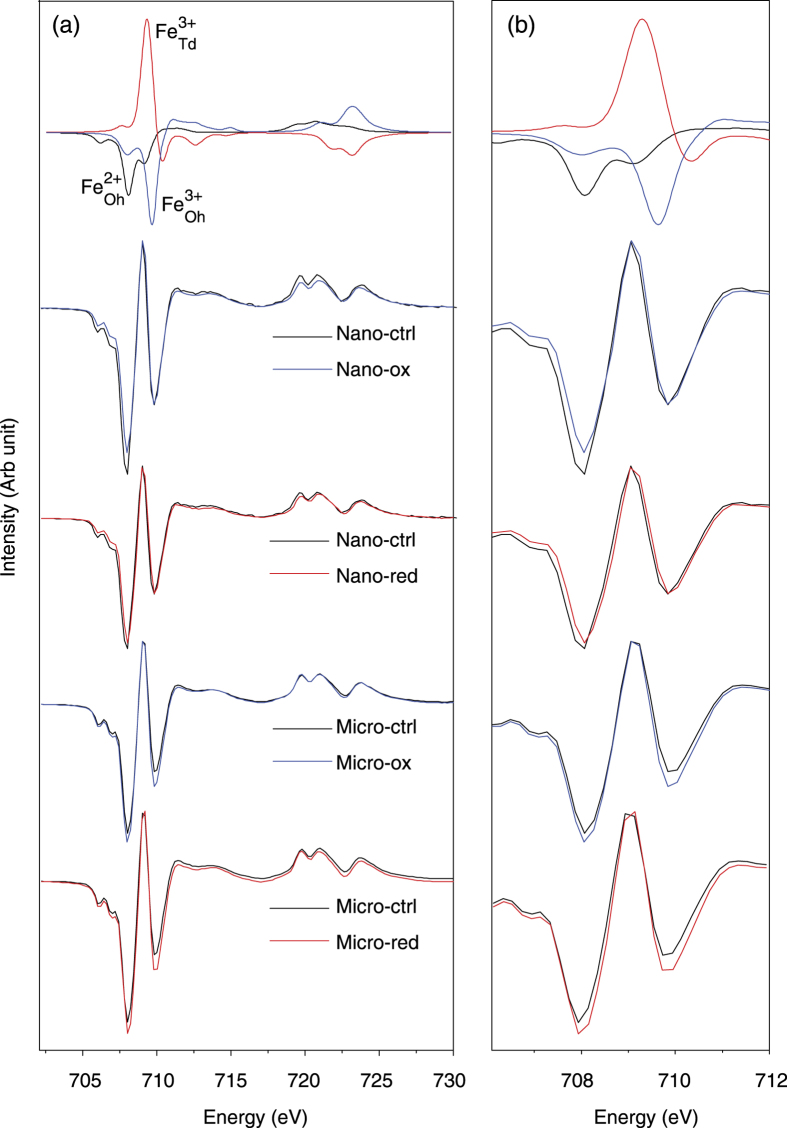
Fe *L*_2,3_ XMCD of Nanomag and Micromag without inoculation (ctrl), after oxidation (ox) and after reduction (red) compared to stoichiometric magnetite. (**a**) *L*_2,3_ edge, (**b**) *L*_3_ edge expanded. The top row shows the individual fits corresponding to the Fe^2+^_Oh_, Fe^3+^_Td_ and Fe^3+^_Oh_ lattice sites for stoichiometric magnetite.

Following the completion of MS experiments, samples were dried for *ex situ* mineralogical analysis using XMCD and Mössbauer spectroscopy to more effectively understand the changes induced by the bacteria. XMCD spectra (Fig. 2) were collected in total electron yield mode, which has a probing depth of 4.5 nm into the material. The characteristic XMCD spectrum at the Fe *L*_2,3_ for magnetite arises due to the antiparallel arrangement of Oh- and Td-coordinated Fe in magnetite, giving rise to three distinct peaks at the *L*_3_ edge. These peaks correspond to the relative abundance of Fe(II) and Fe(III) oxidation states and their coordination environment, with the lowest energy negative peak matching Fe^2+^_Oh_, the positive peak matching Fe^3+^_Td_ and the second negative peak matching Fe^3+^_Oh_ 25. The XMCD data obtained before and after oxidation/reduction are shown in Fig. 2, with the spectra normalized to the Fe^3+^_Td_ site because it is generally the most stable iron site in the magnetite structure, with each displaying the characteristic signature expected for magnetite^26^. It is evident from Fig. 2, that the only clear differences between Nano-ctrl and Nano-ox are the intensity of the Fe^2+^_Oh_ peak (708 eV), in which the intensity of Nano-ctrl is greater than that of Nano-ox. However, in the case of Micromag it appears as though the intensity of the Fe^3+^_Oh_ and Fe^2+^_Oh_ peaks have increased after oxidation (Micro-ox) in comparison to the starting material (Micro-ctrl). There were no obvious differences between the spectra of Nano-ctrl and Nano-red which suggests that no reduction of the Nanomag took place despite the evidence to the contrary shown by MS and Mössbauer (see below) data. The relative intensity of both octahedral Fe sites appears to increase for the microparticles, relative to Fe^3+^_Td_.

Based on fitting theoretical component spectra from atomic multiplet calculations, the relative intensity of each lattice site was determined (Table 1). In all cases, the intensity of Fe^2+^_Oh_ is far greater than would be expected of stoichiometric magnetite (Fe(II)/Fe(III) = 0.5), with the Fe(II)/Fe(III) ratio ranging from 0.64 to 0.75 across the samples. This suggests that all of the magnetite nanoparticles investigated were highly reduced at their surfaces. Such a result has previously been observed for magnetite particles by Liu *et al*.^9^ and is attributed to spontaneous enrichment of Fe(II) electron density above bulk stoichiometry from particle interiors to the mineral surface while in contact with dilute aqueous solution. The exponentially decreasing probing depth for XMCD measured by total electron yield is 4.5 nm, so that 50% of the signal comes from the top 3.11 nm and 90% of the signal from the top 10.36 nm of the sample^27^. So for these materials XMCD can still be considered a surface sensitive measurement. Both Nanomag and Micromag were put into a medium containing phosphate and other ions, which could have contributed to this enhanced distribution at the surface of the particles. Despite this, the trends supporting the microbial oxidation of both Nanomag and Micromag by *R. palustris* can be observed, with Fe(II)/Fe(III) decreasing from 0.75 to 0.64 for Nanomag, and decreasing from 0.75 to 0.70 for Micromag. There was little difference observed between the Nano-ctrl and Nano-red XMCD spectra, though a decrease in Fe(II)/Fe(III) from 0.75 to 0.73 respectively was calculated which in fact suggests some oxidation. The Fe(II)/Fe(III) ratios of Micro-ctrl and Micro-red were calculated to be 0.75 and 0.71 which would also suggest some oxidation.

**Figure 3:**
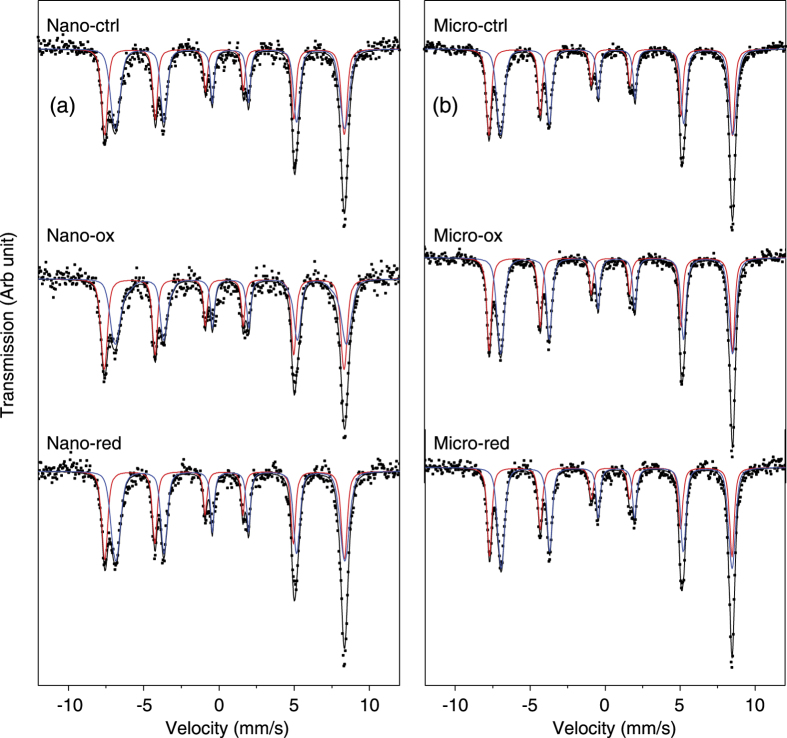
Mössbauer spectroscopy at 140 K of (**a**) Nanomag and (**b**) Micromag. Red sextets correspond to tetrahedral (Td) coordinated iron, with blue sextets corresponding to octahedral (Oh) coordinated iron. Raw data is represented by filled dots, with the sum of the fits shown as a black line.

Bulk sensitive ^57^Fe Mössbauer spectroscopy was performed on all six samples at 140 K (Fig. 3). All spectra display the characteristic overlapping two sextets characteristic of magnetite corresponding to Td and Oh sites respectively^28^. No other Fe(III) or Fe(II) mineral phases were observed. The hyperfine parameters of each fit are shown in Table 2, none of which appear to show differences with each other in either the Nanomag or Micromag sample series which indicates that the fundamental mineralogy of both Nanomag and Micromag remains essentially the same even after either oxidation or reduction. The relative populations of each of the Td and Oh were used to determine the stoichiometry of the samples^29^ (Table 2). 140 K was chosen as the most suitable temperature for this calculation based on the fact that all samples will be well below their blocking temperature, whilst simultaneously above the Verwey transition (~121 K)^29^.

The results indicate that the starting materials are initially oxidized in their interiors with an Fe(II)/Fe(III) ratio of 0.40 and 0.41 for Nanomag and Micromag, respectively, consistent with the observed Fe(II) enrichment at surfaces deduced from XMCD spectroscopy. Microbial Fe(II) oxidation of the nanoparticles leads to a notable decrease in the bulk Fe(II)/Fe(III) ratio, whilst reduction leads to a slight increase, following the expected trends. For Micromag, however, Fe(II)/Fe(III) ratio of the oxidized material remains largely unchanged (within error). The reduced microparticles show a small but significant increase, which is as expected. Attempts to calculate the extent of Fe(II) oxidation based on Mössbauer Fe(II)/Fe(III) ratios have been made (see Supplementary Note 1). These calculations show that the Nanomag and Micromag starting materials are 16% and 14% oxidized respectively. Nano-ox shows a clear increase in the extent of oxidation, whilst the Nano-red shows only a small decrease in extent of oxidation which is with the range of error. The effect of microbial Fe(II) oxidation on Micromag appears to be within error, whilst Micro-red shows a decrease in the extent of Fe(II) oxidation which is significant.

### Magnetometry measurements

**Figure 4:**
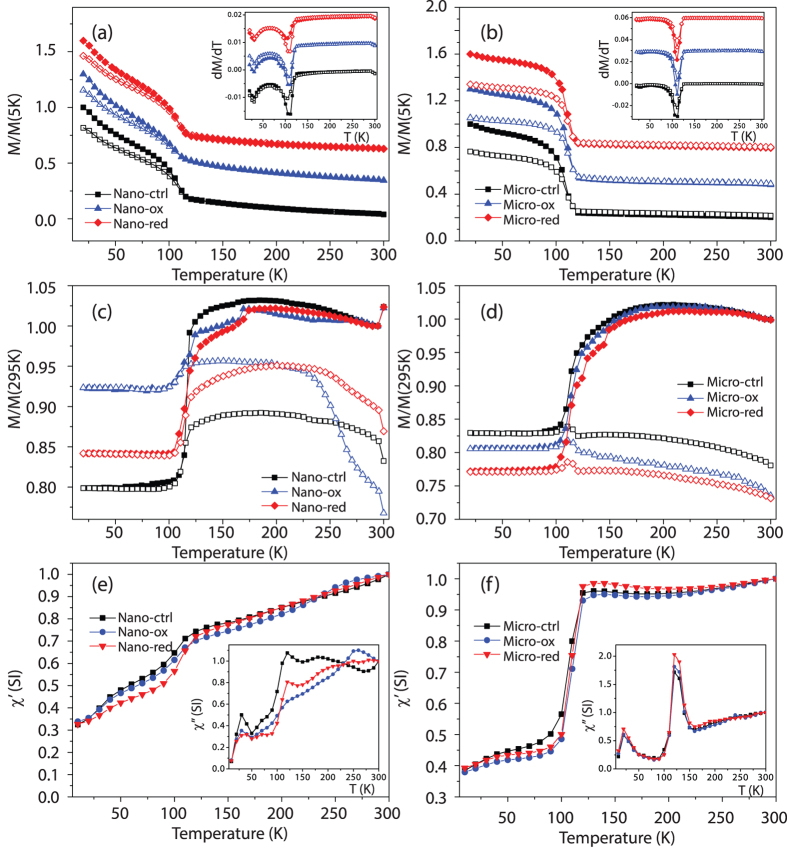
Magnetic characterization – (**a**) FC-ZFC-saturation isothermal remanent magnetization and first order derivative of FC-ZFC-SIRM (inset figure) of Nanomag, (**b**) FC-ZFC-SIRM and first order derivative of FC-ZFC-SIRM (inset figure) of Micromag. In (**a,b**) solid and hollow symbols represent FC (ZFC) data during warming and cooling respectively. (**c**) Room temperature (RT-SIRM) of Nanomag, (**d**) RT-SIRM of Micromag. In (**c,d**) solid and hollow symbols show magnetization measured on cooling and warming respectively. (**e**) In-phase (χ′) and quadrature (inset figure; χ″) AC-frequency dependent susceptibility of Nanomag, (**f**) In-phase (χ′) and quadrature (inset figure; χ″) AC-frequency dependent susceptibility of Micromag.

Further analysis of the changing properties of the magnetite induced by microbial interaction was carried out using low temperature superconducting quantum interference device (SQUID) magnetometry in conjunction with field-cooled and zero-field-cooled saturation magnetisation isothermal remanence magnetisation (FC-ZFC-SIRM) and room temperature SIRM (RT-SIRM) experimental protocols. Furthermore, Alternating Current (AC) field dependent measurements were also collected (Fig. 4).

The FC-ZFC-SIRM of both Nanomag and Micromag series exhibit characteristic behaviour of magnetite. The dramatic change in slope observed in both sets of curves is associated with the Verwey transition (*T*_V_)^30^. The FC magnetization is higher than the corresponding ZFC magnetization between 20 K and 120 K for all nano- and micro-particle samples, which is consistent with populations of grains dominated by single-domain-like behaviour at low temperature. The first-order derivative of the FC-ZFC-SIRM curves were used to estimate *T*_V_ (inset figures, Supplementary Table 1). The values in Supplementary Table 1 are taken from the average of the values determined from the FC and ZFC derivatives. It can be seen that *T*_V_ is approximately 4 K lower for Nano-ox than both Nano-ctrl and Nano-red, which suggests a degree of maghemitization^23^. In the case of Micromag, *T*_V_ appears to increase after both oxidation and reduction, though the increase is smaller than the step size of the measurement, and hence below the resolution of the data set. The remanence ratios, *δ,* (defined in Supplementary Table 1) of the nanoparticles are close to 1.3, compared to 1.4 for the microparticles. In comparison, magnetite magnetosomes internalised within magnetotactic bacteria are noted to have a remanence ratio above 2.0^31^, especially if they are partially maghemitized^32^. The lower *δ* values associated with the synthesized magnetites in this study are likely due to magnetostatic interactions within aggregates of clumped grains.

RT-SIRM curves show differences due to oxidation/reduction (Fig. 4c,d). This is especially true for Nano-ox in which the warming curve magnetization falls off dramatically and continues to fall to 80% of *M*_s(295)_. This behaviour is not observed in either Nano-ctrl or Nano-red and is likely related to the effect of oxidation^23^. In-phase AC susceptibility (χ′) curves (1 Hz) do not show any major differences in either the Nanomag or Micromag series (Fig. 4e,f) and yield nearly identical data when collected at 10 and 100 Hz (data not shown). The quadrature susceptibility (χ″) of Nano-ox does show strikingly different behaviour to that of both Nano-ox and Nano-red, with a clear absence of a peak close to *T*_V_, which is present in the other samples. χ″ of the microparticles do not show clear differences.

The hysteresis loops (Supplementary Figure 1) are characteristic of pure magnetite, and are notable in that the Nanomag particles have almost no coercivity (*H*_c_) in comparison to the larger microparticles. Given the mean diameter of the Nanomag particles (12 nm), one would expect to see room temperature hysteresis loops dominated by superparamagnetic behaviour, whereas the larger mean grain sizes in the Micromag sample are expected to show more elevated coercivities and discernable hysteresis. The saturation magnetisation values (*M*_s_) of the starting materials are close to the theoretical value of 92 A m^2^ kg^−1^ expected of magnetite at room temperature. The slightly smaller value observed for Nano-ctrl could again indicate that the sample is slightly oxidized, or due to the effect of its small particle size^33^.

Decreases in *M*_s_ were observed for Nano-ox (−23%) and Micro-ox (−9%) compared to their respective starting materials. For the reduction experiments, the *M*_s_ for Nano-red decreased (−13%) which is suggestive of an oxidation process, whereas Micro-red *M*_s_ increased by (+6%). Very small differences were observed between the coercivities of the nanoparticle and microparticle series.

### Electron Microscopy

**Figure 5:**
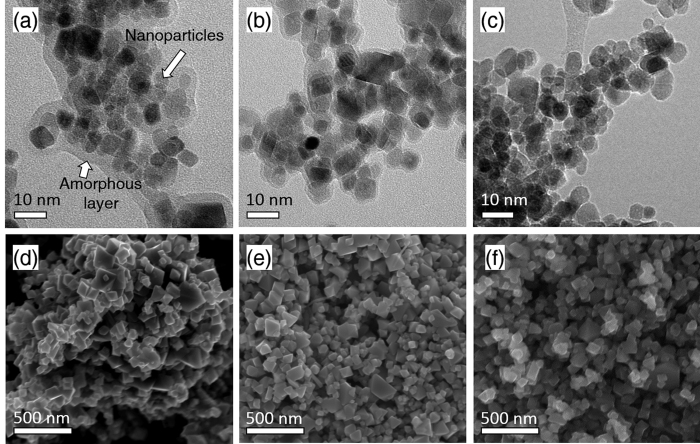
Electron microscopy. Transmission electron microscopy (TEM) of samples (**a**) Nano-ctrl, (**b**) Nano-ox and (**c**) Nano-red. Scanning electron microscopy (SEM) of samples (**d**) Micro-ctrl, (**e**) Micro-ox and (**f**) Micro-red.

Both Nanomag and Micromag were imaged with TEM and SEM respectively. In each of the Nanomag series, an amorphous layer appeared to be present around all samples (Fig. 5a–c) and thought to form during sample preparation^34^. Previous TEM imaging on the same material which had not been added to media did not show this amorphous layer^35^. Despite this, all samples show relatively similar shaped particles (i.e. round with some squared particles). Analysis of the particle size indicates little differences between the samples with Nano-ctrl having average *d* = 12.3 nm (σ = 0.3 nm) and Nano-ox having *d* = 12.0 nm (σ = 0.3 nm), however, Nano-red appeared to show a slight increase in particle size in comparison to the other samples with *d* = 13.3 nm (σ = 0.3 nm). This is somewhat contradictory to previous studies, which suggest that *Geobacter sulfurreducens* can lead to the dissolution of magnetite^11^,^36^, although it is possible that such a dissolution process might take place if the reduction phase was permitted to proceed over a longer timescale. The grain size distributions of the microparticles also do not appear to show much difference from each other, with the particle size averaging between 100–200 nm for all samples in the series (Fig. 5d–f).

## Discussion

### Surface vs bulk reactions

**Figure 6:**
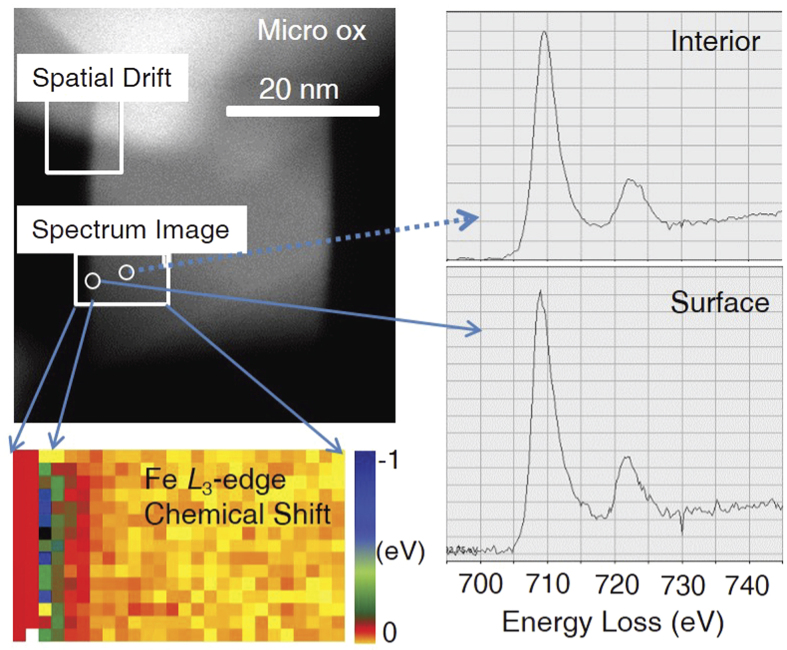
Scanning transmission electron microscopy-high angle annular dark field (STEM-HAADF) image and STEM electron energy-loss spectroscopy (EELS) imaging that identifies the region of high Fe(II) with respect to the core for the Micro-ox sample. The top left corner is the STEM-HAADF image on which the STEM-EELS mapping region and a drift correction region were marked. Bottom left is the Fe *L*_3_-edge chemical shift map, indicating the Fe *L*_3_-edge captured from the surface of the particle shows an obvious negative chemical shift, indicating the Fe at the particle surface is more reduced than that in the inner part of the particle. The right panel is a comparison of the EELS spectra from the surface and interior of the particle, with data collection locations as indicated by the arrows.

One of the most striking results of this study is the difference in stoichiometry of both the Nanomag and Micromag starting materials observed by XMCD compared to Mössbauer spectroscopy. The amount of Fe(II)/Fe(III) ratio determined by XMCD (*x* ≈ 0.64–0.75) was far in excess of the expected stoichiometric value of 0.5. In comparison, the Mössbauer data indicated that both nanoparticles and microparticles were slightly oxidized (*x* ≈ 0.38–0.43). The XMCD signal is surface sensitive with an exponentially decreasing probing depth of 4.5 nm, whilst Mössbauer works via transmission and will probe the entire crystal. This suggests that the surfaces of the particles are highly enriched in Fe(II) compared to their cores. Scanning transmission electron microscopy–high angle annular dark field (STEM-HAADF) mapping was applied on Micro-ox, because their larger crystal sizes were more accessible to this technique, and confirmed such a surface layer to be present (Fig. 6). This is in agreement with previous studies that show a tendency for magnetite nanoparticles in aqueous solution to spontaneously segregate electron density to the particle-solution interface, and that this tendency can persist through redox reactions arising from changes in solution conditions or solutes. For example, oxidation by acidic dissolution through dilution of the initial particle suspensions (~pH 8) in NaHCO_3_ buffer (pH 7) produces an oxidized interior (as shown by XRD data) while the surface region remains enriched in Fe(II) relative to Fe(III) (as shown by XMCD data)^9^.

With subsequent oxidation by *R. palustris* TIE-1, both XMCD and Mössbauer support oxidation of the mineral phases, in line with previous studies^8^,^9^,^13^, with the changes more pronounced in the XMCD measurements. This enables us to conclude that the microbial Fe(II) oxidation is highly dependent upon the surface properties of the particles, and it is thus likely that surface stoichiometry will play a significant role in the ability for Fe(II)-oxidizing bacteria to oxidize magnetite. This hypothesis is further supported by the fact that the difference is more pronounced in the case of the nanoparticles than in the microparticles, which highlights the importance of the surface area to volume ratio effect. However, in the case of reduction, the results of both Mössbauer and XMCD are less clear despite the fact that it has previously been shown that bacteria are capable of reducing magnetite^10^,^11^,^13^,^37^. The XMCD data suggest that the Fe(II)/Fe(III) ratio at the particle surface is very similar if not slightly oxidized after exposure to the Fe(III)-reducing bacteria. However, using Mössbauer spectroscopy, it could be seen that the Fe(II)/Fe(III) ratio for the whole magnetite particle did in fact increase as a result of microbial Fe(III) reduction. These differences between the XMCD and Mössbauer suggest that the surfaces of the particles remain relatively unmodified by the bacteria, whilst particle cores are reduced, consistent with some type of electron transport through the surface into the mineral lattice^35^.

The high abundance of surface Fe(II) in these synthetic magnetites is likely one of the reasons why the Fe(II)-oxidizing and Fe(III)-reducing bacteria behave so differently with respect to the surface and the bulk. As the ratio of Fe(II)/Fe(III) determined using XMCD (i.e., the ratio at the magnetite surface) appears to be high, it is possible that the magnetite surfaces are saturated with a supply of Fe(II), which the Fe(II)-oxidizer *R. palustris* TIE-1 directly accesses and converts to Fe(III); this simultaneous accumulation of an Fe(III) product at the surface results in a larger net decrease in the surface Fe(II)/Fe(III) ratio, whilst the core remains relatively intact. The surface saturation with Fe(II), however, may also hinder the electron transfer by Fe(III)-reducing bacteria as the surfaces of the magnetite particles are already so highly reduced that any further reduction of the surface is not or hardly possible. Instead, we suggest that an electron transport process must be enlisted to enable reduction to proceed, transferring charge through the surface via an electron hopping mechanisms to the core of the magnetite and leading to the overall reduction of the core of the particles, a process that could manifest the observed changes to the magnetic susceptibility.

### Magnetic properties

The low temperature magnetic properties of the samples reveal broad similarities between the reduced and starting materials as compared to the oxidized samples. The most notable changes occur in the RT-SIRM data for Nano-ox. Whilst the initial change in remanence magnetization is the smallest for Nano-ox samples on cooling, the warming curve shows dramatic differences to the other samples. These data suggest that low-temperature magnetic methods can distinguish magnetite particles which have undergone oxidation (through either microbial or abiotic pathways) from those that have not been oxidized. However, the reduction of both magnetite nanoparticles and microparticles does not yield significant differences in low temperature magnetic properties, which potentially rules it out as a diagnostic tool for such processes. Notwithstanding, it is evident that reduction does lead to significant changes to the room temperature volume dependent magnetic susceptibility (Fig. 1b), which could potentially have a profound impact on the use of this technique for recording changes to the magnetic content of soils and sediments. Changes in susceptibility might not simply be confined to the principle of increased or decreased magnetic material, but may also be a reflection of microbial processes that are currently poorly understood (see recent review by Maxbauer *et al*. (2016)^38^. An increasing number of studies suggest that bacteria have a direct influence on magnetic properties of soils and sediments^39^, and low temperature magnetometry appears to be one of the more useful and sensitive characterization tools in this line of study^40^. Thus, there is a close link between microbiology and geophysics, specifically environmental- and rock magnetism, and even simple metrics such as magnetic susceptibility may provide an important signature that could be used as an indicator for redox conditions in the past and present^41^,^42^.

### Environmental implications

The results presented here have several implications in terms of the interactions of bacteria with magnetite and other redox active Fe minerals in the environment. Perhaps most important is the relevance to the processes by which long range electron transport is mediated by extracellular electron transfer. For instance, the different methods through which Fe(III)-reducing bacteria are able to use solid electron acceptors, such as ferrihydrite, require either direct contact (operating over distances <1.8 nm), the production of chelators (nm-μm), use of electron shuttles (nm-μm), or the formation of nanowires, which are appendages that appear to facilitate electron transport over several micrometers. However, recently a number of different studies have attempted to learn more about these types of long range electron transport^43^ and also the potential role of iron minerals (including magnetite) in facilitating it^44^. A recent study suggested that sulfide minerals can even enable centimetre-long electron transport in marine sediments^45^. Our results support the idea that mixed valent iron minerals, such as magnetite, can potentially play a much more important role in electron transfer than previously considered. In the context of microbial Fe(II) oxidation, it is clear that surface stoichiometry plays a major role in the ability of Fe(II)-oxidizers to use magnetite as an electron donor, and that oxidation is perhaps unlikely to occur in locations where abiotic oxidation is able to induce maghemitization of the mineral surface. By contrast, we observe that microbial Fe(III) reduction can take place regardless of the size of the magnetite (i.e., microparticles and nanoparticles can both be relatively easily reduced), and that any large magnetite crystals could potentially be used as a terminal electron acceptor for Fe(III)-reducing bacteria.

Finally, these findings also have implications for the use of magnetite, predominantly in the form of nanoparticles, for environmental remediation strategies of waste water and contaminated soils and sediments^46^. Specifically, nano-magnetite has been previously shown to react with a variety of heavy metals (e.g., Cr and U) as well as other compounds (e.g., ArNO_2_), making it a suitable candidate for environmental remediation^14^,^15^,^47^,^48^. However, the reactivity of magnetite is highly dependent upon its Fe(II) content, with reduction rates significantly inhibited by decreasing Fe(II)/Fe(III) stoichiometry^16^,^17^. Thus, the microbial oxidation or reduction of magnetite demonstrated in this study could significantly impact the redox reactivity at the mineral surface and directly influence its remediation capabilities^49^.

## Conclusions

We have shown that magnetite can undergo microbial redox processes as both nanoparticles and microparticles. Whilst microbial Fe(II) oxidation appears to be highly sensitive to the surface area to volume ratio of the magnetite, this relationship does not appear to be valid for microbial Fe(III) reduction. Instead we suggest that microbial Fe(III)-reducers drive an electron hopping mechanism through the particles, thus rendering it a bulk dependent effect. Such differences between surface/bulk processes could potentially have significant impact on (*i*) the identification of biomarkers and changes to redox conditions in the rock record using magnetic signatures; (*ii*) the suitability of magnetite in mediating long range intercellular electron exchange within soils and sediments; (*iii*) use of magnetite as a remediation agent for treating wastewater or contaminated soils and sediments.

## Materials and Methods

### Magnetite synthesis

Nanomag was synthesised at room temperature using the protocol outlined in Pearce *et al.*^35^. In brief, an anoxic solution of FeCl_2_ (1 M) and FeCl_3_ (2 M) in HCl (0.3 M) was added dropwise into an anoxic solution of NH_4_OH (25%) under continuous stirring at 800 rpm in an anoxic glovebox (100% N_2_). This process led to the instantaneous precipitation of magnetite nanoparticles (d ≈ 12 nm), which were washed twice in ultrapure deionized water to remove chloride ions. The point of zero charge was determined to be pH 6-7 for the starting material and the long term stability of stoichiometry was determined via micro x-ray diffraction (μ-XRD) and X-ray magnetic circular dichroism (XMCD)^13^.

Micromagnetite particles were prepared following the modified method of Schwertmann and Cornell^50^ in which the starting FeSO_4_ solution was stored in the presence of zerovalent Fe for one month prior to reaction to ensure no Fe(III) was present. Subsequent oxidation of the FeSO_4_ in an alkaline solution of KNO_3_ at 90 °C under a N_2_ atmosphere yielded the magnetite particles, which were dried and stored under N_2_ until analysis. Analysis following new hybrid oxidi-colorimetric method Amonette and Matyáš^51^ revealed that micromagnetite was close to stoichiometric (~92%) with a composition of 70.96 ± 0.41 wt% Fe_Total_ and 21.84 ± 0.23 wt% Fe(II).

### Bacteria growth conditions

*Rhodopseudomonas palustris* strain TIE-1 was grown on modified basal medium^5^ containing aqueous Fe(II) as an electron donor incubated under constant light (True Light 15 W/5500 K). Prior to the experiment two generations were cultivated on Fe-free media containing 10 mM Na-acetate as electron donor to ensure no residual iron was present. The strict anaerobe *Geobacter sulfurreducens* was cultivated on modified fresh water medium^52^ with 25 mM Na-acetate as electron donor and 40 mM Na-fumarate as electron acceptor. Cultures of *R. palustris* and *G. sulfurreducens* were grown without Fe until the late log phase after which they were washed by repeated cycles of centrifugation and resuspension in anoxic NaHCO_3_ buffer (30 mM; pH 7). After final centrifugation dense cell suspensions were prepared for each strain in 7.5 ml and 7.0 ml for *R. palustris* and *G. sulfurreducens,* respectively.

### Experimental set-up

Experiments were prepared in 50 ml headspace vials containing 25 ml of modified basal medium, pH 7, NaHCO_3_ buffer. Aliquots of magnetite (10 mg) were added to bottles from stock solutions and allowed to equilibrate for 3 days. Acetate (1 mM) was added to all reduction experiment bottles. Dense cell suspensions of either *R. palustris* or *G. sulfurreducens* were added into the bottles, equivalent to 100% (v/v) inoculum. Fe(II) oxidation experiments were incubated in the light at 20 °C for 48 h until changes in magnetic susceptibility no longer appeared to decrease. Fe(III) reduction experiments were incubated in the dark at 30 °C for 24 h, at which point magnetic susceptibility data showed no significant increase and the experiments were terminated to prevent potential mineral dissolution^11^,^36^. During the incubations, magnetic susceptibility was the only parameter that was monitored.

### Mineralogical analysis

For synchrotron-based XMCD measurements, dried magnetite samples were loaded onto carbon tape, which was fixed to a sample holder. All sample preparation was carried out in an anoxic glovebag (100% N_2_) to prevent abiotic oxidation. The samples were loaded into the Portable Octupole Magnet System (POMS) on beamline I10 at the Diamond Light Source^53^ in a backflow of nitrogen and then held in a vacuum for the duration of the measurement. Samples were exposed to circularly polarized x-rays across the energy range of the Fe *L*_2,3_ edge (690–740 eV) with spectra recorded using total electron yield (TEY) detection in energy steps of 0.2 eV with an effective probing depth of ~45 Å 27. XMCD data were obtained from the difference between x-ray absorption (XAS) spectra collected under opposite magnetic fields (±0.5 T). XMCD data were interpreted using a non-linear least squares approach, with each site corresponding to Fe^2+^_Oh_, Fe^3+^_Td_ and Fe^3+^_Oh_, where Oh and Td denote octahedral and tetrahedral coordination, respectively, fitted against calculated spectra^54^,^55^ with the 10*Dq* crystal-field parameters taken as 1.4 and −0.7 eV for Fe Oh and Td sites, respectively. The results were convoluted by a Lorentzian with a half-width at half-maximum of *Γ* = 0.3 (0.5) eV for the *L*_3_ (*L*_2_) edge to account for intrinsic core-hole lifetime broadening and by a Gaussian of *σ* = 0.2 eV to account for instrumental broadening.

Mössbauer spectroscopy was carried out at the University of Tübingen, with samples prepared in a glovebox (100% N_2_) by mixing the dried powder (~10 mg) with glucose (80 mg) and ground using a pestle and mortar to ensure a homogenous sample. Magnetite-glucose mixtures were loaded into Plexiglas holders (area 1 cm^2^) and then loaded into a closed-cycle exchange gas cryostat (Janis cryogenics) under a backflow of He. Spectra were collected at 140 K with a constant acceleration drive system (WissEL) in transmission mode with a ^57^Co/Rh source and calibrated against a 7 μm thick α-^57^Fe foil measured at room temperature. Spectral fitting was carried out using an Voigt based fitting (VBF) routine in the Recoil software (University of Ottawa)^56^. The half width at half maximum (HWHM) was constrained to 0.133 mm/s during fitting.

Scanning transmission electron microscopy images (STEM) and electron energy loss spectroscopy (EELS) were carried out using aberration corrected electron microscope, including JEOL JEM-ARM200CF microscope operated at 200 kV and FEI Titan 80–300 operated at 300 kV. Both microscopes are equipped with a probe spherical aberration corrector and post column attached with a Gantan Quantum EELS system for spectroscopic imaging. For the STEM-high angle annular dark field (HAADF) imaging, the inner and outer collection angles of annular dark field detector were set at 68 and 280 mrad, respectively. For EELS spectroscopic imaging, the energy resolution is typically in the range of 0.8 eV. The high resolution TEM image were collected using FEI Titan 80–300 operated at 300 kV and fitted with an aberractor for objective lens. The average particle size was determined by measuring the longest length of individually resolvable particles and a population size of *n* ≈ 150 for each sample.

Scanning electron microscopy (SEM) analysis was performed to analyse particle morphology amongst control and reacted, reduced and oxidized, magnetite samples. Samples were prepared for imaging by fixation of magnetite powders to carbon tape and carbon coating with a thickness of ~10 nm. SEM imaging was performed on Quanta 3D FEG, equipped with an energy-dispersive X-ray detector for qualitative analysis at acceleration voltage of 5 to 10 keV.

### Magnetic measurements

*In-situ* magnetic susceptibility was measured on triplicate experiments at room temperature with a KLY-3 Kappabridge (AGICO, Czech Republic), at a peak magnetic field of 300 A m^−1^ and a frequency of 875 Hz. Bottles (50 ml headspace vials) containing the magnetite, media and bacteria were loaded directly into the instrument for each measurement. The susceptibility of the input vessel was subtracted from the final value to account for diamagnetic signal of the glass and media. The T-test function was applied to magnetic susceptibility data using two tails and two-sample unequal variance (i.e., type 3). The test was applied to experiments carried out in triplicate, with direct comparisons made between Nano-ox:Micro-ox, Nano-red:Micro-red, Nano-ox:Nano-ctrl, Micro-ox:Micro-ctrl, Nano-red:Nano-ctrl and Micro-red:Micro-ctrl. A value of P < 0.05 is considered to be statistically significant.

Low temperature magnetic measurements were carried out on all of the dried magnetite samples at the Institute for Rock Magnetism, University of Minnesota, USA. Samples were transported in individual anoxic bags, which were only opened within a glovebox (COY; 95% N_2_, 5% H_2_). Powdered sample were put between two layers of quartz wool packing material and sealed inside pharmaceutical gelatine capsules, which were then sealed with Kapton tape to maintain anoxic conditions. The capsules were transported to the instrument within N_2_ filled Eppendorf vials and only removed immediately prior to loading into an instrument. Several low temperature magnetic measurements were performed using a Quantum Design MPMPS-2 superconducting quantum interference device (SQUID) magnetometer, including (*i*) field-cooled (FC) and zero-field-cooled (ZFC) low temperature saturation isothermal remanence magnetization (LTSIRM) sweep curves which were obtained by applying 2.5 T during cooling from 300 K to 20 K with FC remanence magnetization measured during warming from 20 K to 300 K in the absence of an applied field. The samples were then cooled to 20 K in the absence of a field, magnetized by a field of 2.5 T, which was switched off before warming the sample to 300 K during which ZFC remanence magnetization measurements were recorded in steps of 5 K. First order differentiation was performed on FC and ZFC curves for all samples to more effectively identify sudden changes in the remanence magnetization curves. (*ii*) Room temperature saturation isothermal remanence magnetization (RT-SIRM) curves were obtained by first applying a magnetic field of 2.5 T at 300 K, which was switched off immediately prior to measuring the magnetization during thermal cycling to 20 K and back to 300 K. (*iii*) In-phase (χ′) and quadrature phase (χ″) frequency dependent magnetic susceptibility measurements were performed at 1, 10 and 100 Hz.

Room temperature hysteresis loops and backfield curves were obtained using a Princeton Measurements Vibrating Sample Magnetometer (VSM) with applied magnetic fields between −1 T and 1 T.

## Additional Information

**How to cite this article**: Byrne, J. M. *et al.* Size dependent microbial oxidation and reduction of magnetite nano- and micro-particles. *Sci. Rep.* **6**, 30969; doi: 10.1038/srep30969 (2016).

## Supplementary Material

Supplementary Information

## Figures and Tables

**Table 1:** Fitting results from XMCD data, normalised to the relative abundance of Fe^3+^_Td_, for Nanomag and Micromag before inoculation (ctrl), after oxidation (ox) and after reduction (red) compared to stoichiometric magnetite (Fe_3_O_4_).

	Fe^2+^_Oh_	Fe^3+^_Td_	Fe^3+^_Oh_	Fe(II)/Fe(III)	Td/Oh
*Fe_3_O_4_*	*1.00*	*1.00*	*1.00*	*0.50*	*0.50*
Nano-ctrl	1.44	1.00	0.93	0.75	0.42
Nano-ox	1.26	1.00	0.96	0.64	0.45
Nano-red	1.41	1.00	0.93	0.73	0.43
Micro-ctrl	1.33	1.00	0.77	0.75	0.48
Micro-ox	1.30	1.00	0.87	0.70	0.46
Micro-red	1.33	1.00	0.88	0.71	0.45

The errors associated with the fitting of each site are estimated to be ±0.02^57^.

**Table 2:** Hyperfine parameters obtained through fitting of Mössbauer data for Nanomag and Micromag series obtained at 140 K via Voigt based fitting^56^.

Sample	Site	CS (mm/s)	ε (mm/s)	H(T)	Pop.	±	Fe(II)/Fe(III)	±
Nano-ctrl	Td	0.37	0.00	49.4	42.7	2.1	0.40	0.02
	Oh	0.75	−0.01	47.2	57.3	2.1		
Nano-ox	Td	0.35	−0.01	49.3	48.6	1.9	0.35	0.02
	Oh	0.79	0.02	47.5	51.4	1.9		
Nano-red	Td	0.35	−0.01	49.3	40.9	1.3	0.42	0.02
	Oh	0.76	0.01	47.2	59.1	1.3		
Micro-ctrl	Td	0.36	0.00	50.1	41.7	0.9	0.41	0.01
	Oh	0.76	0.00	47.8	58.3	0.9		
Micro-ox	Td	0.37	0.01	50.1	40.9	0.8	0.42	0.01
	Oh	0.75	0.00	47.8	59.1	0.8		
Micro-red	Td	0.36	0.01	50.1	38.9	1.1	0.44	0.01
	Oh	0.75	0.00	47.8	61.1	1.1		

Td corresponds to tetrahedral coordinated sextets and Oh corresponds to octahedral coordinated sextets. CS = center shift, ε = quadrupole shift, H = hyperfine field, Pop = relative abundance.
