# Supplementary material for: Size dependent microbial oxidation and reduction of magnetite nano- and micro-particles
Source: Sci Rep. 2016 Aug 5;6:30969. doi: 10.1038/srep30969 (PMC4974511; doi:10.1038/srep30969)
Supplement: Supplementary Information [file srep30969-s1.pdf]

Size dependent microbial oxidation and reduction of magnetite nano- and micro-particles

James M. Byrne[1], Gerrit van der Laan[2,3], Adriana I. Figueroa[2], Odeta Qafoku[4], Chongmin Wang[4], Carolyn I. Pearce[5], Michael Jackson[6], Joshua Feinberg[6], Kevin M. Rosso[4], Andreas Kappler[1]

1. *Geomicrobiology, Center for Applied Geosciences, University of Tuebingen, Sigwartstrasse 10, 72076, Tuebingen, Germany*
2. *Magnetic Spectroscopy Group, Diamond Light Source, Didcot, OX11 0DE, UK*
3. *Williamson Research Centre for Molecular Environmental Science, School of Earth, Atmospheric and Environmental Sciences, University of Manchester, Manchester M13 9PL, UK*
4. *Pacific Northwest National Laboratory, Richland, WA 99352, USA*
5. *School of Chemistry, The University of Manchester, M13 9PL, Manchester, UK*
6. *Institute for Rock Magnetism, University of Minnesota, 291 Shepherd Labs, 100 Union Street SE, Minneapolis, MN 55455*

*corresponding author email: James.Byrne@uni-tuebingen.de

Supplementary Information

Supplementary Figure

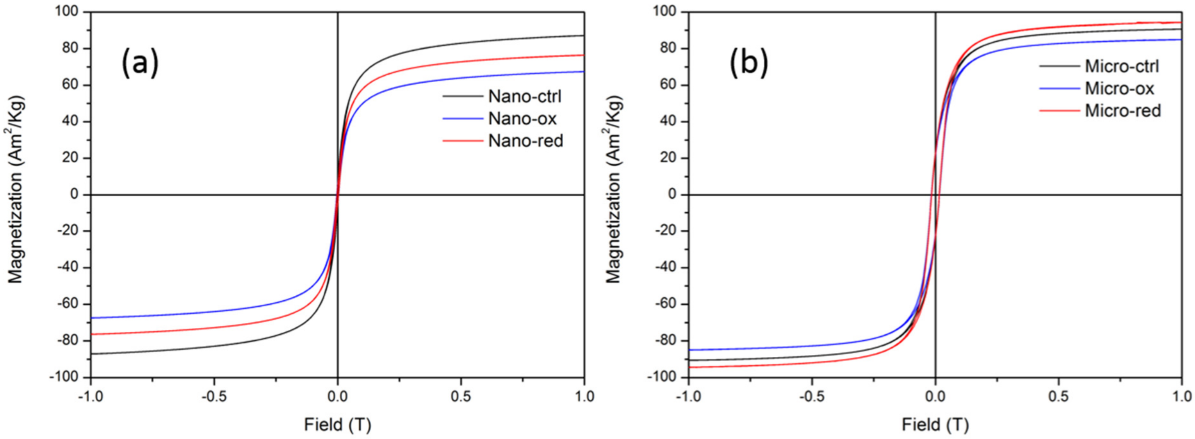

Supplementary Figure 1– Room temperature hysteresis loops obtained using a vibrating sample magnetometer (VSM). (a) Nanomag series, (b) Micromag series.

Supplementary Table

	M_s (A m^2 kg^{-1})	M_r (A m^2 kg^{-1})	B_c (mT)	B_{rh} (mT)	M_r/M_s	B_{rh}/B_c	δ_{FC} (A m^2 kg^{-1})	δ_{ZFC} (A m^2 kg^{-1})	δ	T_V (K)
Nano-ctrl	88.55	6.46	3.34	15.35	0.07	4.60	12.1	9.13	1.33	108.5
Nano-ox	68.53	4.97	3.51	15.80	0.07	4.50	4.37	3.21	1.36	104.2
Nano-red	77.28	4.99	3.11	16.62	0.06	5.34	8.11	6.6	1.23	108.4
Micro-ctrl	92.43	23.40	16.03	29.48	0.25	1.84	21.8	15.4	1.42	108.8
Micro-ox	83.66	22.00	16.14	29.41	0.26	1.82	26.1	17.9	1.46	110.6
Micro-red	97.65	22.80	15.49	31.12	0.23	2.01	26.2	17.6	1.49	110.3

Supplementary Table 1 – Magnetic characteristics. M_s = saturation magnetization at 295 K, M_r = remanent magnetization, B_c = coercivity, B_{rc} = coercivity of remanence, δ_{FC} = relative loss of remanence during field cooling, δ_{ZFC} = relative loss of remanence during zero field cooling, δ = delta ratio (δ_{FC}/δ_{ZFC}), and T_v = Verwey transition temperature.

Supplementary Note

The heterogeneous distribution of Fe(II) which we observe in the magnetite (based on the difference observed between XMCD and Mössbauer data) means that an accurate estimate of how much iron has been reduced or oxidized is difficult to determine. However, it is possible to make a rough estimate using the stoichiometry values determined via Mössbauer spectroscopy as these are representative of the bulk. To couple this number to the evolving stoichiometry of the nanoparticles during oxidation or re-reduction, for charge balance it is necessary to consider loss of Fe atoms and the formation of cationic vacancies in the magnetite (Fe_3O_4) which arise during oxidation. The fully oxidized version of magnetite, known as maghemite, has the formula $(Fe(III)^{Td}[Fe(III)_{5/3}\square_{1/3}]^{Oh}O_4$ where $\square$ represents a vacancy, Td and Oh correspond to tetrahedral and octahedral coordination respectively. We therefore consider the complete formula for magnetite to be $(Fe(III))^{Td}[Fe(II)_{1-3\delta}Fe(III)_{1+2\delta}\square_{\delta}]^{Oh}O_4$. The stoichiometry ($X$) is related to δ by:

$$X = \frac{(1-3\delta)}{(2+2\delta)} \tag{1}$$

which can be rearranged as:

$$\delta = \frac{(1-2X)}{(2X+3)} \tag{2}$$

The % of the magnetite which is occupied by Fe(II) can be determined as:

$$\%Fe(II) = 300\delta \tag{3}$$

Based on equation 3, the relative proportion of Fe(II) which is oxidized (%Fe(II) oxidation) in the Nanomag and Micromag can be determined. However, this is only valid when the Fe(II)/Fe(III) ratio is less than or equal to 0.5, i.e., this can only be applied to Mössbauer data.

	**Fe(II)/Fe(III) (Mössbauer)**	**±**	**δ**	**%Fe(II) oxidation**	**±**
Theory	0.50	0	0	0	0
Nano-ctrl	0.40	0.02	0.05	15.8	1.7
Nano-ox	0.35	0.02	0.08	24.3	1.8
Nano-red	0.42	0.02	0.04	12.5	1.6
Micro-ctrl	0.41	0.01	0.05	14.1	0.8
Micro-ox	0.42	0.01	0.04	12.5	0.8
Micro-red	0.44	0.01	0.03	9.3	0.8

Supplementary Table 2 – Calculated magnetite stoichiometric (Fe(II)/Fe(III)) ratios based on Mössbauer spectroscopy measurements.

From Supplementary Table 2, it can be seen that degree of oxidation in Nanomag increases after exposure to oxidizing bacteria, whilst for the Micromag the degree of oxidation in Micro-ox is within error of the starting material (Micro-ctrl). After reduction, there is a decrease in the degree of oxidation in both Nano-red and Micro-red in comparison to their respective starting values, although the decrease for Nano-red is within error of Nano-ctrl. Based on this information, the data appears to suggest that changes within the Fe(II)/Fe(III) ratio were within error for the case of Nanomag reduction and Micromag oxidation. As the XMCD results indicate a significant amount of Fe(II) is present at the surface of the particles it is not possible to perform such estimates for them.
